# Assessing Awareness, Attitude, and Practices of Breast Cancer Screening and Prevention Among General Public and Physicians in Pakistan: A Nation With the Highest Breast Cancer Incidence in Asia

**DOI:** 10.1155/2024/2128388

**Published:** 2024-09-28

**Authors:** Adeel Aslam, Asma Ghulam Mustafa, Ali Hussnain, Hafsa Saeed, Fatima Nazar, Maha Amjad, Ayesha Mahmood, Atika Afzal, Anam Fatima, Doaa Kamal Alkhalidi

**Affiliations:** ^1^ Faculty of Pharmacy and Biomedical Sciences Mahsa University, Jenjarom, Selangor, Malaysia; ^2^ Department of Pharmacy University of Lahore, Lahore, Punjab, Pakistan; ^3^ Pharmacy Practice Department Dubai Pharmacy College for Girls, Dubai, UAE

**Keywords:** breast cancer attitude, breast cancer Pakistan, breast cancer practices, general population awareness regarding breast cancer

## Abstract

**Introduction:** Breast cancer is a global health challenge with significant mortality, affecting millions worldwide. The current study is aimed at evaluating awareness and practices related to breast cancer screening, prevention, and treatment among the general public and physicians in Lahore, Pakistan, which has a significant incidence of breast cancer.

**Methodology:** The current study adopted a cross-sectional study design conducted in Lahore, Pakistan, between March and August 2023, among 404 participants from the general public and 240 physicians. Data collection and evaluation involved the use of validated questionnaires, and both descriptive and inferential statistics were performed using SPSS Version 25.

**Result:** In Lahore, Pakistan, breast cancer awareness among the public was low, with 80.2% unaware of its global prevalence, 65.3% believing not everyone is at risk, and only 42.1% recognizing symptoms. Females showed greater awareness (OR: 1.020, CI: 0.617–1.686, *p* = 0.002) and positive attitudes (OR: 2.711, CI: 1.478–6.478, *p* = 0.045), while the 18–29 age group had higher odds of positive practices (OR: 4.317, CI: 2.678–5.956, *p* = 0.004). Educational attainment significantly influences knowledge and attitudes. Only 13.9% practiced self-examination. Among physicians, 88.8% were confident in screenings, but patient fear (42.9%) and financial barriers (79.2%) hindered action. Physicians with FCPS qualifications had higher odds of awareness (OR: 1.550, CI: 1.130–2.117, *p* = 0.007), attitudes (OR: 1.500, CI: 1.050–2.150, *p* = 0.025), and practices (OR: 1.470, CI: 1.070–2.017, *p* = 0.020). Those with 11–20 years of experience also showed better awareness (OR: 1.400, CI: 1.050–1.868, *p* = 0.022) and attitudes (OR: 1.450, CI: 1.045–2.018, *p* = 0.029).

**Conclusion:** In conclusion, breast cancer awareness among the general public is limited, highlighting the need for tailored education programs. Although most physicians show high awareness, challenges in patient communication and barriers, such as fear and financial constraints, must be addressed to improve screening uptake. These findings emphasize the importance of targeted interventions to enhance public awareness, screening practices, and physician-patient communication.

## 1. Introduction

Breast cancer is a malignant growth that originates in the lining cells of the ducts or lobules within the glandular tissue of the breast [1]. Breast cancer is a global health challenge that affects millions of women and, in some cases, men across the world [2]. It is the most commonly diagnosed cancer among women and is the leading cause of cancer-related deaths worldwide [3]. In 2020, 685,000 people died worldwide from breast cancer, with 2.3 million new cases diagnosed [4]. Breast cancer is the most common cancer worldwide, with 7.8 million people surviving as of the end of 2020 who had been diagnosed within the previous five years [5]. The burden of breast cancer transcends geographical boundaries, impacting both developed and developing countries [6]. Because breast cancer is diagnosed and treated earlier in developed nations, the prognosis and survival rates are better [7]. The majority of patients with advanced or metastatic breast cancer appear in low-resource nations, with poor prognosis [8]. Among Asian countries, Pakistan has the highest rate of breast cancer incidence; with one in nine women being diagnosed with the disease [9, 10]. As per the 2018 report from the International Agency for Research on Cancer, there were 34,066 new cases of breast cancer recorded in Pakistan [11]. The risk factors contributing to the development of breast cancer are multifaceted, encompassing aging, genetic mutations, early onset of menstruation, delayed or no pregnancies, experiencing menopause after the age of 55, lack of physical activity, postmenopausal weight gain or obesity, having dense breast tissue, using combination hormone therapy, oral contraceptives, personal history of breast cancer or certain noncancerous breast conditions, family history of breast cancer, previous radiation therapy, and alcohol consumption [12, 13]. In response to the prevalence and significance of breast cancer, several screening methods have been developed to detect and diagnose the disease effectively. [14]. These include breast self-examination (BSE), clinical breast examination (CBE), and mammography [15]. Unlike CBE and mammography, which require a doctor's visit, BSE can be performed at home on their own [16]. This procedure is simple, noninvasive, and takes only 5 min [17]. Small tumors are more likely to be detected early in breast cancer since the disease progresses gradually, and early diagnosis increases the likelihood of a better prognosis and more effective therapy [18]. The survival rate for breast cancer diagnosed at Stages 1 and 2 is 85%, but it drops significantly to 10% for late-stage diagnoses, specifically Stage 4 [19]. Therefore, the early detection and management of breast cancer are pivotal in improving patient outcomes and reducing mortality [20]. Despite high breast cancer incidence rates in Pakistan, there is a significant gap in understanding the specific aspects of awareness, attitudes, and practices related to breast cancer screening and prevention. Previous studies have not adequately explored detailed knowledge, specific attitudes, or practical behaviors, nor have they addressed how demographic factors like gender, age, education, and family history influence these aspects. Pakistani society, characterized by its diverse cultural, religious, and socioeconomic dynamics, presents a unique backdrop for examining the awareness and responses to breast cancer [21]. The prevailing attitudes toward health, traditional beliefs, and healthcare infrastructure often significantly influence individuals' understanding of, and engagement with, breast cancer-related issues [22]. Within the complex web of factors influencing breast cancer outcomes, the role of physicians is pivotal [23]. As the primary gatekeepers of medical knowledge and patient care, physicians play a critical role in the early detection, treatment, and overall management of breast cancer cases [24]. This study is aimed at filling these gaps by evaluating breast cancer-related knowledge, attitudes, and practices among the general public in Lahore, including men and women, as well as physicians. Previous studies have fallen short by not including physicians, whereas our study incorporates the perspectives of both the public and physicians, providing a more comprehensive understanding. Furthermore, no previous studies in Pakistan have included both men and women in their assessment; our study fills this gap by encompassing both genders and also integrating the insights of physicians. The objective of this study is to assess the level of awareness, attitude, and practices regarding breast cancer screening, prevention, and treatment among the public and physicians of Pakistan, a nation with the highest breast cancer incidence in Asia.

## 2. Materials and Methods

### 2.1. Study Design

A cross-sectional survey-based study design using a quantitative approach was employed for the current study.

### 2.2. Study Setting

The study was conducted between March and August 2023 to assess the awareness, attitude, and practices regarding breast cancer among the general population and physicians in Lahore, Pakistan's second-largest city [25]. Data was collected from the outside of major hospitals in Lahore. The data from physicians was gathered directly from hospitals. Physicians had the option to select more than one practice setting if they were involved in multiple roles, such as working part time in a clinic, health center, or private practice. This allowed for the inclusion of diverse practice environments and ensured a comprehensive representation of their professional settings.

### 2.3. Study Population and Sample Size

The current study comprised participants from both the general population and physicians (including general practitioners and oncologists) who were 18 years and older. The inclusion of the male general population in this study was designed to provide a more comprehensive understanding of breast cancer awareness and attitudes across all segments of the community. Although the incidence of breast cancer in men is considerably lower, it is important to gauge their awareness and involvement in breast cancer-related discussions, particularly as they may play supportive roles for female relatives or friends affected by the disease. The study objectives were clearly communicated to potential participants, who willingly agreed to participate by signing an informed consent form. Participation was entirely voluntary, and no incentives were provided. Individuals failing to meet the specified inclusion criteria were excluded from the study. Moreover, those who declined to provide informed consent or submitted incomplete questionnaires were also excluded. Trained research staff was available to assist participants with no formal education in understanding and completing the questionnaires. This approach ensured that all participants could contribute regardless of literacy levels. The sample size was determined with a 95% confidence level using Rasoft software, indicating a minimum requirement of 385 subjects from each population. The main focus was on collecting data from 500 respondents; however, 54 participants did not provide consent, and 42 questionnaires were incomplete. As a result, 404 fully completed questionnaires were included. Additionally, we aimed to survey 450 respondents from physicians, but only 300 consented. Due to the hospital's approval for a limited 6-month data collection period, 240 questionnaires were fully completed and included in the current study. The discrepancy between the calculated minimum sample size of 385 physicians and the actual number of 240 respondents arose due to a limited 6-month data collection period, challenges in accessing a large number of physicians, and delays in obtaining consent. The final sample size consisted of 404 respondents from the general public and 240 respondents from physicians.

### 2.4. Study Instrument

The study's data were gathered using questionnaires that were created after conducting a thorough review of existing literature [24, 26–28], and questionnaires were tailored separately for physicians and the general public. Prior to commencing the primary study, a pilot study was conducted. Both questionnaires demonstrated a satisfactory level of internal consistency, as evidenced by their respective Cronbach's alpha values. Cronbach's alpha for Questionnaire 1 (general population) was calculated to be 0.736, While for Questionnaire 2 (physicians), it was 0.689. In addition to internal consistency, construct validity was assessed, with Questionnaire 1 scoring 0.87 and Questionnaire 2 scoring 0.79, affirming the reliability and consistency of the questionnaire's results. The final version of the questionnaire comprised four sections, encompassing sociodemographic characteristics, knowledge, attitudes, and practices related to breast cancer withall sections consisted of close-ended questions. Questionnaire 1 was also translated into Urdu as well.

### 2.5. Sampling Technique and Data Collection

Data from the general public were collected using a convenient sampling technique, because it was practical and easy to access a diverse sample quickly. Additionally, conducting surveys in Lahore, a city in the Islamic Republic of Pakistan, presented challenges, particularly when asking sensitive questions to female participants. For physicians, a snowball sampling technique was employed, which relied on existing professional networks to recruit participants despite their busy schedules. These methods were chosen to maximize participation and gather comprehensive data within the time and budget limits. The confidentiality and anonymity of all participants were strictly maintained throughout the study.

### 2.6. Data Analysis

Immediately after collecting the data, it was evaluated and categorized through assessment and tagging processes. Subsequently, it was coded and entered into the Statistical Package for Social Sciences (IBM® SPSS) Version 25. The analysis involved both descriptive and inferential statistics. Descriptive statistics, including frequencies and percentages, were used to summarize the data. Inferential statistics utilized chi-square test, with the significance level set at *p* ≤ 0.05. The findings are presented in tables and figures. For the awareness and knowledge of breast cancer, results were categorized into good and poor knowledge. A score of 1 was assigned to each correct answer, while “0” was assigned for each incorrect answer. Participants scoring ≥ 50% were classified as having good knowledge, while those with scores < 50% were categorized as having poor knowledge. The practices of breast cancer screening techniques were similarly categorized into poor and good practices based on the same classification used for knowledge of BSE. For a more in-depth analysis, logistic regression was applied to compute odds ratios (ORs), along with their respective 95% confidence intervals (CIs). Initially, 1 point was assigned for each correct answer, while 0 points were assigned for each incorrect response. All correct answers were then individually introduced into bivariate logistic regression models to evaluate their association with sociodemographic characteristics.

### 2.7. Ethical Considerations

The study received ethical approval from the University of Lahore (IREC/2022/17-H), and the research was carried out in full compliance with the ethical guidelines established by the institutional research committee.

## 3. Result

### 3.1. General Public

#### 3.1.1. Demographic Characteristics

Table 1 presentsdetailed demographic characteristics of the general public, offering valuable insights into their gender distribution, age demographics, educational backgrounds, and family history of breast cancer. Among the general public, 68.3% were female, 29.5% were male, and 2.2% chose not to disclose their gender preference. The age distribution showed a clear majority (68.8%) in the 18-29 age group, followed by 30-39 (16.3%), 40-49 (7.9%), 50-59 (4.5%), and aged 60 and above (2.5%). Educational backgrounds varied, with a significant proportion of participants having completed a graduate degree (59.9%), followed by intermediate (23.0%), a master's degree (9.7%), and individuals with no formal education (7.4%). Regarding family medical history, 91.1% of respondents reported no family history of breast cancer, while 8.9% acknowledged a family history of the disease.

#### 3.1.2. Awareness, Attitude, and Practices

Table 2 outlined key responses from respondents addressing breast cancer awareness and preventive practices. A substantial portion of participants, 80.2%, were not aware that breast cancer ranked as the most common cancer among women worldwide. An equal percentage lacked knowledge about the associated risk factors. While 34.7% believed everyone is at risk for breast cancer, a majority, 65.3%, held a contrary view. Notably, 42.1% could identify symptoms of breast cancer, but only 13.9% had performed BSE. Awareness of BSE as a screening method was limited to 27.2%. Although 60.1% believed breast cancer could be prevented, disparities were evident in encouraging others for screening, with only 41.6% expressing willingness. Furthermore, just 8.9% had undergone a mammogram, and 5.2% had a CBE by a healthcare professional. The data emphasize the need for enhanced education and advocacy to bridge knowledge gaps and promote proactive measures for breast health within communities.

**Figure 1:**
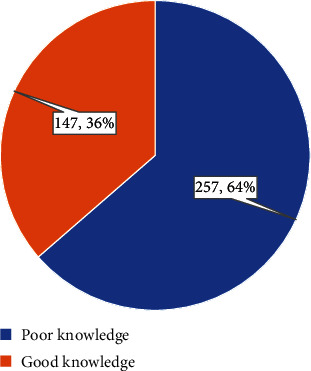
Level of knowledge and awareness regarding breast cancer among the general public.

The assessment of respondents' knowledge and awareness of breast cancer revealed that only 147 (36%) had a good level of knowledge, while 257 (64%) demonstrated poor knowledge (Figure 1).

**Figure 2:**
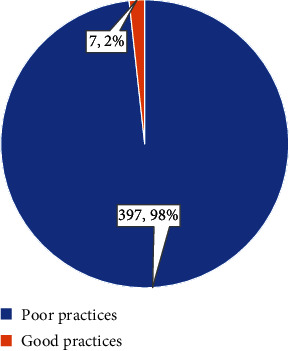
Level of practice of breast cancer screening techniques among the general public.

The level of practice of breast cancer screening techniques among respondents is depicted in Figure 2. The results indicated that 397 (98%)participants had a poor level of practice, while only 7 (2%) participants exhibited good practices.

#### 3.1.3. Adherence to the Screening Guidelines

The respondents who said that they performed the BSE, CBE, and mammogram were further asked how frequently they performed.  Table 3 provides a comprehensive overview of adherence to breast cancer screening guidelines across various age groups. For monthly BSE, the highest adherence was observed in the 18–29 age group (33.9%), with a gradual decrease in older age categories. Annually, CBE displayed varied adherence patterns, with the highest percentage in the 40–49 age group (33.3%). Annually, mammogram adherence follows a distinct trend, with the 40–49 age group exhibiting the highest percentage (38.1%). Notably, adherence to these screening methods tended to decrease in the older age groups. The table offered valuable insights into the screening practices among different age demographics, emphasizing potential areas for targeted interventions to enhance breast cancer awareness and early detection within the general population.

#### 3.1.4. Factors Influencing Breast Cancer Screening Practices

Table 4 presented a comprehensive overview of factors influencing breast cancer screening practices, categorizing responses based on three key screening methods BSE, CBE, and mammography. Notably, 22% of participants cited having no breast problems as a reason for not engaging in BSE, while 33.1% found the practice embarrassing. For CBE, 21.2% emphasize a lack of breast issues, with 44.3% finding the examination embarrassing. In the case of mammography, 13.1% indicated no breast problems, while 50.4% expressed embarrassment as a deterrent. Fear of diagnosis was acknowledged by 6.4%, 4.9%, and 4.45% for BSE, CBE, and mammography, respectively. The fear of radiation or the procedure was minimal, primarily affecting CBE (2.7%) and mammography (2.73). A substantial portion, 14.8%, admitted to not considering screening as necessary for BSE, contrasting with 9.9% for mammography. Lack of time was a common barrier, affecting 12.1%, 13.3%, and 12.6% for BSE, CBE, and mammography, respectively. Finally, 24.7% of male participants acknowledged that they did not see the need for breast screening practices. These findings underscored the diverse array of factors influencing individuals' decisions regarding breast cancer screening, emphasizing the need for tailored interventions to address specific concerns and barriers within the population.

#### 3.1.5. Regression Analysis Between Demographics, Knowledge, Attitude, and Practice of Breast Cancer Among Study Participants

In Table 5, OR with their corresponding 95% CIs presented to evaluate the association between gender, age, education, and family history of breast cancer with knowledge, attitude, and practices related to breast cancer screening and prevention. Regarding gender, the an OR of 1.020 (95% CI: 0.617–1.686) and a significant *p* value of 0.002 indicated that females were more likely to have increased knowledge, while an OR of 2.711 (95% CI: 1.478–6.478) and a *p* value of 0.045 suggested that they exhibited more positive attitudes. For the age variable, statistically significant ORs and *p* values across age groups highlighted the age-specific nuances in breast cancer awareness.The 18–29 age group demonstrating a higher likelihood of positive practices (OR: 4.317, 95% CI: 2.678–5.956, *p* value: 0.004), suggesting greater engagement in preventive behaviors. Furthermore, educational attainment played a crucial role, with graduates exhibiting significantly elevated ORs for both knowledge and attitudes. Additionally, individuals with a family history of breast cancer showed a substantially increased likelihood of engaging positive practices (OR: 0.384, 95% CI: 0.023–0.764, *p* value: 0.023). These findings highlight the importance of tailoring public health interventions to demographic characteristics n order to improve breast cancer awareness and promote preventive practices among the general public.

### 3.2. Physicians

#### 3.2.1. Demographic Characteristics

Table 6 provided detailed demographic characteristics of the physicians, offering valuable insights into their age demographics, educational backgrounds, designation, experience, and workplace locations. In terms of age distribution, the majority fell within the age range of 25–35 years (44.5%), followed by those between 36 and 45 years (23.7%). Education levels were predominantly represented by individuals with MBBS degrees (82.0%), with a smaller percentage holding MD (2.0%) and FCPS (16.0%) qualifications. The designation was mostly composed of general practitioners (94.5%), with a minority being oncologists (5.5%). In terms of experience, over half of the professionals had less than 5 years of experience (52.9%), while 40.4% had 5–10 years. The place of work varied,with the majority employed in hospitals (65.0%), followed by clinics (11.2%), health centers (12.0%), and private practices (11.6%).

#### 3.2.2. Awareness Related to Breast Cancer

Table 7 Provided a comprehensive insight into breast cancer awareness among physicians, focusing on known risk factors, signs, symptoms, and diagnostic methods. A substantial majority of physicians correctly identified age (45%), smoking (87.5%), high-fat diet (37%), alcohol consumption (22.9%), and genetic factors (89.1%) as established risk factors for breast cancer development. In recognizing signs and symptoms, the majority correctly identified a lump in the breast (93.3%), a change in breast size or shape (87.5%), and breast pain (90.4%). However, awareness awareness varied for nipple discharge (64.4%) and skin dimpling (44.5%). Regarding diagnostic methods, an overwhelming percentage correctly acknowledged the role of mammography (98.7%), biopsy (87%), and breast MRI (90%), while ultrasound was recognized by 82.5%. This comprehensive understanding among physicians was crucial for ensuring early detection and effective management of breast cancer, emphasizing the need for ongoing education and awareness initiatives in areas where knowledge gaps existed.

**Figure 3:**
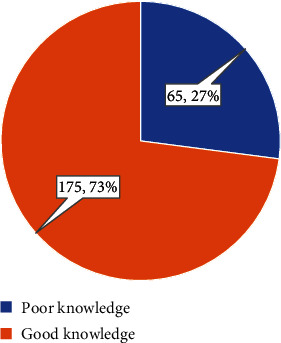
Level of knowledge of breast cancer screening techniques among physicians.

The level of knowledge of breast cancer screening techniques among physicians is depicted in Figure 3. The results indicated that the majority, 175 (73%), had a good level of knowledge, while 65 (27%) demonstrated poor knowledge.

#### 3.2.3. Attitude Toward Breast Cancer Screening

Table 8 presents a detailed overview of physicians' attitudes toward breast cancer screening. A majority strongly agreed (80.4%) that breast cancer screening was essential for early detection, reflecting widespread acknowledgment of its importance. Furthermore, a high level of confidence was observed, with 88.8% expressing confidence in their ability to conduct breast cancer screenings. The commitment to staying informed was also evident, as 86.2% reported regularly updating their knowledge of breast cancer screening guidelines. Despite this positive outlook, a significant proportion (82.1%) found it challenging to convince patients to undergo regular breast cancer screening, highlighting a potential barrier in the healthcare communication process. These findings offered valuable insights into physicians' perspectives, pointing toward both strong support for the significance of screening and potential areas for intervention to improve patient engagement in screening practices.

#### 3.2.4. Practices Related to Breast Cancer

Table 9 details physicians' practices related to breast cancer screening, encompassing the types of screenings recommended, the age at which recommendations commenced, the frequency of recommendations, and the educational approaches employed. The majority consistently advocated for BSE (23.8%), CBE (83.8%), mammography (98.8%), and MRI (87.5%). Age-wise, most recommended starting screenings between the ages of 25 and 40 (80.4%), with some variations in recommendations across different age groups . The frequency of screenings also varied, with a significant proportion advising monthly screenings (23.8%) and annual screenings (91.2%). The frequency of recommendations varied, with a significant proportion advising monthly screenings (23.8%) and annual screenings (91.2%). Noteworthy educational practices included verbal advice during consultations (92.9%) and using visual aids/models for demonstration (93.8%). Additionally, distributing printed information (43.3%) and hosting educational workshops (8.3%) were also employed.

**Figure 4:**
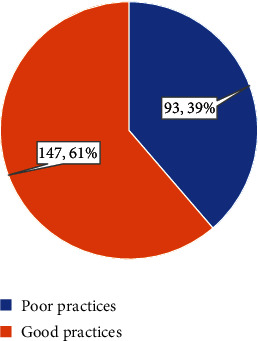
Level of practice of breast cancer screening techniques among physicians.

The level of practice of breast cancer screening techniques among physicians is depicted in Figure 4. The results indicated that the majority, 147 (61%), had a good level of practice, while 93 (39%) demonstrated poor practices.

#### 3.2.5. Barriers Encounter When Recommending Breast Cancer Screenings to Patients

Table 10 elucidates barriers faced by physicians when recommending breast cancer screenings to patients. Patient-related factors such as fear or anxiety were acknowledged by 42.9% of respondents, highlighting the psychological aspect that may hinder screening uptake. Lack of patient awareness is identified as a significant challenge by 82.1%, underscoring the need of educational initiatives. Cultural or social stigma was recognized by 66.2%, indicating the need for culturally sensitive approaches. Financial constraints, including the cost of screening procedures, pose substantial barriers, with 79.2% and 88.8% of respondents acknowledging these challenges, respectively. Additionally, limited access to healthcare facilities was perceived as a barrier by 76.3%, highlighting disparities in healthcare accessibility. These findings collectively emphasize multifaceted challenges, ranging from psychological barriers to financial constraints and healthcare access, warranting comprehensive strategies to overcome these impediments and enhance breast cancer screening adherence among patients.

#### 3.2.6. Regression Analysis

In Table 11, ORs with their corresponding 95% CI are presented to evaluate knowledge, attitudes, and practices related to breast cancer among physicians. Regarding age, physicians aged 46–55 years showed a higher OR for positive attitudes (OR = 1.400, 95% CI: 0.985–1.985, *p* = 0.059), suggesting a trend toward improved attitudes with increased age. In terms of education, physicians with FCPS qualifications had significantly higher ORs for knowledge (OR = 1.550, 95% CI: 1.130–2.117, *p* = 0.007), attitudes (OR = 1.500, 95% CI: 1.050–2.150, *p* = 0.025), and practices (OR = 1.470, 95% CI: 1.070–2.017, *p* = 0.020). Regarding experience, physicians with 11–20 years of experience had significantly higher ORs for knowledge (OR = 1.400, 95% CI: 1.050–1.868, *p* = 0.022) and attitudes (OR = 1.450, 95% CI: 1.045–2.018, *p* = 0.029). These findings underscore the importance of advanced education and professional experience in improving knowledge and attitudes toward breast cancer screening among physicians.

## 4. Discussion

Breast cancer stands as the most prevalent cancer globally. Research consistently supports the fact that early detection and prompt treatment of this disease contribute significantly to reducing mortality rates [29, 30]. In the current research, data were gathered from both the general population and physicians, each group with a distinct background, so their discussions are separated accordingly.

### 4.1. General Public

The findings of this study indicate that the general public in Pakistan, a nation with the highest breast cancer incidence in Asia, possesses relatively limited knowledge about breast cancer. BSE has been identified as an early detection tool for breast cancer. According to the findings of the current study, only a few of the participants (27.2%) were aware of BSE, which is consistent with reports among university students in Ethiopia [31], Nigeria [32], and India [33]. The results also revealed that 56.7% of respondents were aware that early detection of breast cancer significantly improves prognosis and chances of survival. This percentage is higher than the report by Gwarzo et al. among undergraduate students in Buea, where only 37.3% knew about the positive impact of early detection [33]. Another study among rural women in Telangana reported a prevalent low level of BSE knowledge [34]. The current study indicated that 64% had poor knowledge about breast cancer, surpassing the findings of Bellgam and Buowari, where 36.5% exhibited poor knowledge [35]. However, our results are lower compared to a study on college girls in the Udupi district (72.5%) with good knowledge level reported by Shalini et al. [36]. The association between sociodemographic variables and breast cancer knowledge showed that age, educational qualifications, and gender significantly influenced respondents' knowledge. Higher educational qualifications were linked to better knowledge, aligning with Dewi et al.'s findings that well-educated women were more knowledgeable about breast cancer [37].

Similarly, good knowledge of BSE was associated with younger age, consistent with findings in Ghana, where younger students showed higher awareness and knowledge scores [38]. Dadzi and Adam also established a negative relationship between age and BSE practice among women in the Volta region of Ghana [39]. It is not unexpected to observe low knowledge levels among the elderly, given that numerous public education materials are available primarily in English. Consequently, individuals who are illiterate or do not comprehend English may not receive sufficient information [40]. This shows that the younger age group is an important predictor of good knowledge of BSE. Our study found that 98% of respondents had poor practices related to breast cancer screening and prevention, a higher percentage than reported in Malaysia [33]. Lack of knowledge, as noted by Nde et al. [41], Suh et al. [42], and Roetzheim et al. [23], was associated with a low level of breast cancer screening practices. Doshi et al. reported alarmingly low BSE practice among Indian dental students [43]. Major hindrances to BSE, CBE, and mammography included feelings of embarrassment, fear of radiation or procedure, fear of diagnosis, lack of knowledge, and lack of time. Kumar and Kashyap identified lack of knowledge, embarrassment, and fear as reasons for not performing BSE, which is consistent with our study findings [44]. Additionally, Nde et al. [41] and Agbonifoh [45] reported a strong association between a family history of breast cancer and the actual practice of BSE among study participants. Significant associations between knowledge, education level, and BSE practice were also observed in a study conducted among women of reproductive age in Ibadan, Nigeria [46].

### 4.2. Physicians

The knowledge and attitudes of physicians toward breast cancer screening methods play a crucial role in influencing patient practices. The current study's findings revealed that a majority of surveyed professionals exhibited good knowledge and a positive attitude. However, some of them held misperceptions regarding the risk factors, causes, and screening methods, coupled with infrequent engagement in screening practices. These results align with Halmata et al. research, where most physicians were well aware of breast cancer prevention, emphasizing early diagnosis to reduce mortality. They mentioned CBE (74.3%), mammography (71.0%), and ultrasound (27.2%) as screening methods, endorsing BSE as an individual screening tool [47, 48]. A similar study among primary healthcare workers in Diyarbakir, Turkey, also reported high knowledge levels regarding breast cancer [49]. In the current study, 93.3% of physicians acknowledged a lump in the breast as the most recognizable warning sign, consistent with Halmata et al. findings, where 90% of health professionals were aware of this symptom [47]. A study conducted in Bolan Medical Complex, Quetta, Pakistan, focused on the knowledge and perceptions of healthcare staff and medical students about breast cancer screening methods. It revealed that 82% considered mammography an important screening method, and 51.6% agreed on the potential of BSE as a screening method [50]. In the current study results, 98.8% believed mammography was crucial for screening, while 23.8% saw BSE as a screening method for breast cancer. Numerous studies assessing knowledge and practices of breast cancer screening, including those with suboptimal knowledge and attitudes, have been conducted [51, 52]. Abdel-Aziz et al. conducted a study in the Al Hassa region of Saudi Arabia, with 816 participants, evaluating perceived barriers to breast cancer screening. They identified personal fears such as fear of physicians, fear of results, and lack of patient awareness as the main barriers [53]. Financial constraints have been identified as a significant barrier in other developing countries [54, 55]. All these barriers are in line with our research.

## 5. Conclusion

In conclusion, this research in Lahore, Pakistan, sheds light on significant gaps in breast cancer awareness and practices among the general public and physicians. The study reveals suboptimal awareness levels, particularly among the public, emphasizing the need for comprehensive educational interventions. To address these gaps, implementing targeted public health campaigns focused on breast cancer education, and incorporating culturally sensitive materials can overcome barriers such as misinformation. Physicians, while generally knowledgeable, face challenges in patient communication and encounter barriers such as patient fear and financial constraints. For physicians, enhancing training programs that emphasize effective communication strategies and addressing financial constraints could improve patient engagement and adherence to screening guidelines. Additionally, developing frameworks for regular community-based screening programs and support systems for patients could help in bridging the gaps identified in this study.

## 6. Limitations

The study's limitations include potential biases due to the sampling techniques used: convenience sampling for the general public and snowball sampling for physicians. The smaller sample size for physicians (240 vs. the calculated 385) and the inclusion of only Lahore-based participants might affect the generalizability of the findings. Additionally, the study did not compare the knowledge between different physician specialties as initially planned due to consent and scheduling issues. The insufficient number of oncologists (only 13) prevented a comparative analysis of breast cancer knowledge between oncologists and general practitioners. The reliance on self-reported data introduces the potential for recall bias, which could impact the accuracy of the results.

Furthermore, the geographical focus on Lahore, Pakistan, may restrict the generalizability of findings to other regions with distinct sociocultural dynamics. Despite these limitations, the study provides valuable insights into breast cancer awareness and practices, offering a foundation for future research. Future studies should consider longer recruitment periods, explore alternative recruitment strategies to achieve a larger and more representative sample of medical professionals, and expand the geographical scope. By using mixed-method study approaches (e.g., qualitative and quantitative), conducting longitudinal studies, and improving physician-patient communication, future research can address these limitations, leading to more robust and generalizable findings.

## 7. Future Implications

Future research in the field of breast cancer awareness and prevention in Pakistan could explore longitudinal studies to track changes in awareness and practices over time, providing insights into the effectiveness of interventions. To improve physician-patient communication, several innovative strategies can be implemented. Developing and implementing patient-centered communication training programs for healthcare providers can enhance their ability to address patient concerns empathetically and effectively. These programs can include modules on active listening, emotional intelligence, and culturally sensitive communication. Utilizing digital health platforms such as telemedicine and mobile health applications can facilitate regular communication and follow-up, providing patients with easier access to care, virtual consultations, and timely reminders for screenings and appointments. Creating interactive educational tools like apps or online portals where patients can learn about breast cancer, its risk factors, and screening methods can engage patients and improve their understanding. Organizing support groups and workshops for patients and their families to discuss breast cancer awareness, share experiences, and receive guidance from healthcare professionals can help demystify the disease and encourage open communication. Establishing robust feedback mechanisms where patients can share their experiences and suggestions for improving communication with healthcare providers can be used to continuously refine and enhance communication strategies. Offering cultural competence training programs to healthcare providers can help in understanding and respecting the cultural backgrounds of their patients, allowing for tailored communication approaches to meet the specific needs and preferences of different patient groups. By implementing these strategies, healthcare providers can significantly improve their communication with patients, leading to better patient engagement, increased trust, and higher adherence to breast cancer screening and prevention practices.

## Figures and Tables

**Table 1:** Demographic characteristics of the general public.

**Characteristics**	**Variables**	**Frequency**	**Percentage (%)**
Gender	Male	119	29.5%
	Female	276	68.3%
	Do not prefer to say	9	2.2%

Age	18–29	278	68.8%
	30–39	66	16.3%
	40–49	32	7.9%
	50–59	18	4.5%
	60 and above	10	2.5%

Education	No formal education	30	7.4%
	Intermediate	93	23.0%
	Graduate	242	59.9%
	Master's degree	39	9.7%

Do you have a family history of breast cancer?	Yes	36	8.9%
	No	368	91.1%

**Table 2:** Breast cancer awareness and preventive practices among the general public.

**Questions**	**Yes**	**No**
Are you aware that breast cancer is the most common cancer among women worldwide?	80 (19.8%)	324 (80.2%)
Are you aware of the risk factors associated with breast cancer?	80 (19.8%)	324 (80.2%)
Do you think that anyone is at risk for breast cancer?	140 (34.7%)	264 (65.3%)
Can you identify any symptoms of breast cancer?	170 (42.1%)	234 (57.9%)
Can breast cancer be prevented?	243 (60.1%)	161 (39.9%)
Do you think that early detection of breast cancer can significantly improve the prognosis and chances of survival?	229 (56.7%)	175 (43.3%)
Would you encourage others in your family or friend circle to get screened for breast cancer?	168 (41.6%)	236 (58.4%)
Are you aware of BSE as a screening method for breast cancer?	110 (27.2%)	294 (72.8%)
Have you performed BSE	56 (13.9%)	348 (86.1%)
Have you had a Mammogram?	36 (8.9%)	368 (91.1%)
Have you had a CBE performed by a healthcare professional?	21 (5.2%)	383 (94.8%)
Have you ever attended any breast cancer awareness program or health talk?	243 (60.1%)	161 (39.9%)

**Table 3:** Adherence to the screening guidelines.

**Category**	**Age groups**				
	**18**–**29**	**30**–**39**	**40**–**49**	**50-59**	**60 and above**
Monthly BSE (*n* = 56)	19 (33.9%)	14 (25%)	9 (16.1%)	8 (14.3%)	6 (10.7%)
Annually CBE (*n* = 36)	10 (27.7%)	2 (5.5%)	12 (33.3%)	7 (19.4%)	6 (16.7%)
Annually mammogram (*n* = 21)	3 (14.2%)	6 (28.5%)	8 (38.1%)	3 (14.3%)	1 (4.7%)

**Table 4:** Factors influencing breast cancer screening practices.

**Reasons**	**Frequency (percentage %)**		
	**BSE**	**CBE**	**Mammography**
I have no breast problem	89 (22.02%)	86 (21.28%)	53 (13.11%)
Embarrassing	134 (33.16%)	179 (44.31%)	204 (50.49%)
Fear of diagnosis	26 (6.44%)	20 (4.95%)	18 (4.45%)
Fear of radiation or procedure	0	11 (2.72%)	11 (2.73%)
Not aware it was necessary	60 (14.85%)	27 (6.67%)	40 (9.90%)
Lack of time	49 (12.12%)	54 (13.37%)	51 (12.62%)
I'm male	46 (11.38%)	27 (6.67%)	27 (6.68%)

**Table 5:** Regression analysis between demographics, knowledge, attitude, and practice of breast cancer among study participants.

**Variables**	**Knowledge**		**Attitude**		**Practices**	
	**Odds ratio (95% confidence interval)**	**p** ** value**	**Odds ratio (95% confidence interval)**	**p** ** value**	**Odds ratio (95% confidence interval)**	**p** ** value**
Gender						
Male	0.703 (0.614–4.215)	1.03	1.446 (1.214–5.671)	0.474	0.764 (0.641–0.974)	0.964
Female	1.020 (0.617–1.686)	0.002	2.711 (1.478–6.478)	0.045	1.548 (1.261–1.834)	< 0.001
Do not prefer to say	Reference					
Age						
18–29	1.614 (0.390–3.681)	0.327	2.121 (1.784–4.574)	< 0.001	4.317 (2.678–5.956)	0.004
30–39	1.600 (0.355–3.741)	0.509	0.987 (0.874–1.347)	0.958	3.459 (2.488–5.565)	0.318
40–49	0.737 (0.547–3.778)	0.541	0.814 (0.625–1.124)	0.751	2.987 (2.060–4.858)	0.417
50–59	0.769 (0.614–4.328)	0.71	0.765 (0.487–1.547)	0.937	1.713 (00480–2.947)	0.567
60 and above	Reference					
Education						
No formal education	1.000 (0.874–3.471)	0.417	0.347 (0.214–.784)	0.874	0.988 (0.874–1.547)	0.687
Intermediate	0.874 (0.714–2.417)	0.541	0.813 (0.672–1.38)	0.741	1.398 (1.000–4.748)	0.475
Graduate	1.475 (1.147–4.254)	< 0.001	3.417 (2.147–6.217)	0.002	1.547 (1.147–4.875)	0.047
Master's degree	Reference					
Do you have a family history of breast cancer?						
Yes	2.402 (0.761–7.583)	0.039	0.217 (0.147–0.514)	0.859	0.384 (0.023–0.764)	0.023
No	Reference					

**Table 6:** Demographic characteristics of physicians.

**Characteristics**	**Variables**	**Frequency**	**Percentage**
Age	Below 25 years	10	4.16%
	25–35 years	107	44.5%
	36–45 years	57	23.7%
	46–55years	38	15.8%
	55 and above	28	11.6%

Education	MBBS	197	82.0%
	MD	5	2.0%
	FCPS	38	16.0%
	Other	Nil	Nil

Designation	General Practitioner	227	94.5%
	Oncologist	13	5.5%

Experience	Less than 5	127	52.9%
	5–10years	97	40.4%
	11–20 years	10	4.1%
	More than 20 years	6	2.5%

Place of working	Hospital	156	65.0%
	Clinic	27	11.2%
	Health center	29	12.0%
	Private practice	28	11.6%
	Other	Nil	Nil

**Table 7:** Breast cancer awareness among physicians.

**Question**	**Variables**	**Yes**	**No**
Which of the following are known risk factors for developing breast cancer?	Age	108 (45%)	132 (55%)
	Smoking	210 (87.5%)	30 (12.5%)
	High-fat diet	89 (37%)	151 (63%)
	Alcohol consumption	55 (22.9%)	185 (77.1%)
	Genetic factors	214 (89.1%)	26 (10.9%)

Which of the following are signs and symptoms that could indicate breast cancer?	Lump in the breast	224 (93.3%)	16 (6.7%)
	Discharge from the nipple	157 (64.4%)	83 (34.6%)
	Change in breast size or shape	210 (87.5%)	30 (12.5%)
	Skin dimpling	107 (44.5%)	133 (55.5%)
	Breast pain	217 (90.4%)	23 (9.6%)

Which of the following methods are used to diagnose breast cancer?	Mammography	237 (98.7%)	3 (1.3%)
	Ultrasound	198 (82.5%)	42 (82.5%)
	Biopsy	209 (87%)	31 (13%)
	Breast MRI	216 (90%)	24 (10%)

**Table 8:** Attitude toward breast cancer screening among physicians.

**Questions**	**Strongly agree**	**Agree**	**Neither agree nor disagree**	**Disagree**	**Strongly disagree**
Breast cancer screening is essential for early detection.	193 (80.4%)	11 (4.6%)	8 (3.3%)	13 (5.4%)	15 (6.2%)
I feel confident in my ability to conduct breast cancer screenings.	213 (88.8%)	5 (2.1%)	10 (4.2%)	3 (1.2%)	7 (2.9%)
I regularly update my knowledge on breast cancer screening guidelines.	207 (86.2%)	11 (4.6%)	7 (2.9%)	11 (4.6%)	4 (1.7%)
It is difficult to convince patients to undergo regular breast cancer screening.	197 (82.1%)	30 (12.5%)	2 (0.8%)	7 (2.9%)	4 (1.7%)

**Table 9:** Practices related to breast cancer among physicians.

**Questions**	**Variables**	**Yes**	**No**
What types of breast cancer screening do you routinely recommend to your patients?	BSE	57 (23.8%)	183 (76.2%)
	CBE	201 (83.8%)	39 (16.2%)
	Mammography	237 (98.8%)	3 (1.2%)
	MRI	210 (87.5%)	30 (12.5%)

At what age do you usually start recommending these screenings to your patients?	Below 25	103 (42.9%)	137 (57.1%)
	25–40	193 (80.4%)	47 (19.6%)
	40–50	219 (91.2%)	21 (8.8%)
	Above 50	157 (65.4%)	83 (34.6%)

How often do you recommend these screenings to your patients?	Monthly	57 (23.8%)	183 (76.2%)
	Every 3 months	153 (63.8%)	87 (36.2%)
	Biannually	186 (77.5%)	54 (22.5%)
	Annually	219 (91.2%)	21 (8.8%)

What steps do you take to educate patients about the importance of regular breast cancer screening?	Verbal advice during consultations	223 (92.9%)	17 (7.1%)
	Distributing printed information	104 (43.3%)	136 (56.7%)
	Hosting or referring to educational workshops/seminars	20 (8.3%)	220 (91.7%)
	Using visual aids/models for demonstration	15 (6.2%)	225 (93.8%)

**Table 10:** Barriers faced by physicians when recommending breast cancer screenings to patients.

**Barriers**	**Yes**	**No**
Patient's fear or anxiety	103 (42.9%)	137 (57.1%)
Lack of patient awareness	197 (82.1%)	43 (17.9%)
Cultural or social stigma	159 (66.2%)	81 (33.8%)
Financial constraints	190 (79.2%)	50 (20.8%)
Cost of screening procedures	213 (88.8%)	27 (11.2%)
Limited access to healthcare facilities	183 (76.3%)	57 (23.7%)

**Table 11:** Regression analysis of physicians.

**Variables**	**Knowledge**		**Attitude**		**Practices**	
	**Odds ratio (95% confidence interval)**	**p** ** value**	**Odds ratio (95% confidence interval)**	**p** ** value**	**Odds ratio (95% confidence interval)**	**p** ** value**
Age						
Below 25 years	1.250 (0.850–1.855)	0.236	1.350 (0.900–2.015)	0.137	1.180 (0.850–1.640)	0.313
25–35 years	1.080 (0.785–1.485)	0.650	1.220 (0.845–1.762)	0.292	1.080 (0.785–1.487)	0.650
36–45 years	1.220 (0.900–1.661)	0.198	1.150 (0.805–1.640)	0.438	1.210 (0.890–1.649)	0.211
46–55years	1.350 (0.965–1.887)	0.075	1.400 (0.985–1.985)	0.059	1.290 (0.965–1.728)	0.076
55 and above	Reference					
Education						
MBBS	1.050 (0.750–1.468)	0.763	1.050 (0.720–1.584)	0.778	1.060 (0.760–1.484)	0.724
MD	1.200 (0.890–1.623)	0.236	1.180 (0.840–1.648)	0.324	1.140 (0.820–1.582)	0.425
FCPS	1.550 (1.130–2.117)	0.007	1.500 (1.050–2.150)	0.025	1.470 (1.070–2.017)	0.020
Other	Reference					
Designation						
General Practitioner	1.080 (0.795–1.463)	0.623	1.100 (0.785–1.546)	0.570	1.120 (0.800–1.581)	0.503
Oncologist	Reference					
Experience						
Less than 5	1.150 (0.850–1.557)	0.366	1.200 (0.880–1.645)	0.229	1.180 (0.860–1.616)	0.307
5–10years	1.200 (0.900–1.605)	0.211	1.250 (0.910–1.716)	0.174	1.210 (0.880–1.664)	0.217
11–20 years	1.400 (1.050–1.868)	0.022	1.450 (1.045–2.018)	0.029	1.350 (0.995–1.832)	0.055
More than 20 years	Reference					
Place of working						
Hospital	1.130 (0.850–1.515)	0.381	1.200 (0.870–1.654)	0.273	1.140 (0.825–1.580)	0.392
Clinic	1.080 (0.800–1.451)	0.620	1.100 (0.780–1.568)	0.583	1.070 (0.770–1.490)	0.674
Health center	1.150 (0.850–1.556)	0.361	1.180 (0.840–1.651)	0.328	1.140 (0.810–1.605)	0.435
Private practice	1.200 (0.900–1.603)	0.219	1.250 (0.910–1.710)	0.180	1.210 (0.880–1.664)	0.217
Other	Reference					

## Data Availability

The data underlying this article will be shared at a reasonable request by the corresponding author.
